# Toward the Development of Data Governance Standards for Using Clinical Free-Text Data in Health Research: Position Paper

**DOI:** 10.2196/16760

**Published:** 2020-06-29

**Authors:** Kerina H Jones, Elizabeth M Ford, Nathan Lea, Lucy J Griffiths, Lamiece Hassan, Sharon Heys, Emma Squires, Goran Nenadic

**Affiliations:** 1 Population Data Science Medical School Swansea University Swansea United Kingdom; 2 Brighton and Sussex Medical School Brighton United Kingdom; 3 Institute of Health Informatics University College London London United Kingdom; 4 Division of Informatics Imaging & Data Sciences University of Manchester Manchester United Kingdom; 5 Department of Computer Science University of Manchester & The Alan Turing Institute Manchester United Kingdom

**Keywords:** ethical, legal, social implications, public engagement, free-text data, information governance

## Abstract

**Background:**

Clinical free-text data (eg, outpatient letters or nursing notes) represent a vast, untapped source of rich information that, if more accessible for research, would clarify and supplement information coded in structured data fields. Data usually need to be deidentified or anonymized before they can be reused for research, but there is a lack of established guidelines to govern effective deidentification and use of free-text information and avoid damaging data utility as a by-product.

**Objective:**

This study aimed to develop recommendations for the creation of data governance standards to integrate with existing frameworks for personal data use, to enable free-text data to be used safely for research for patient and public benefit.

**Methods:**

We outlined data protection legislation and regulations relating to the United Kingdom for context and conducted a rapid literature review and UK-based case studies to explore data governance models used in working with free-text data. We also engaged with stakeholders, including text-mining researchers and the general public, to explore perceived barriers and solutions in working with clinical free-text.

**Results:**

We proposed a set of recommendations, including the need for authoritative guidance on data governance for the reuse of free-text data, to ensure public transparency in data flows and uses, to treat deidentified free-text data as potentially identifiable with use limited to accredited data safe havens, and to commit to a culture of continuous improvement to understand the relationships between the efficacy of deidentification and reidentification risks, so this can be communicated to all stakeholders.

**Conclusions:**

By drawing together the findings of a combination of activities, we present a position paper to contribute to the development of data governance standards for the reuse of clinical free-text data for secondary purposes. While working in accordance with existing data governance frameworks, there is a need for further work to take forward the recommendations we have proposed, with commitment and investment, to assure and expand the safe reuse of clinical free-text data for public benefit.

## Introduction

### Background

Structured electronic health records (EHRs) have long been used in large-scale research to create new knowledge to inform clinical care, practice, and policy. There are many enterprises specializing in making EHR data available in accordance with jurisdictional legislation and governance [1-4]. We refer to structured data as information recorded in specified forms and fields within the EHRs. Generally, clinical data are highly personal and sensitive and, therefore, need to be deidentified or anonymized before they can be used for any secondary purposes outside the clinical environment. We refer to deidentified data as records from which the commonly recognized identifiers (eg, name, address, and date of birth) have been removed and anonymized data as information from which the data subject cannot be identified [5]. EHR data also contain free-text components, including notes made at consultations and referral letters, which, by definition, are unstructured. As such, they tend to be less available beyond the immediate setting and are typically not readily available for secondary uses such as research or service improvement. However, clinical free-text data represent a vast, untapped source of rich information to guide research and clinical care, including patient-specific context and details that clarify and supplement information coded in structured data fields. Furthermore, some clinical information in mental health, pathology, and imaging reports is not available in coding structures but only in free-text form. By their nature, free-text data are highly likely to contain sensitive information and patient identifiers, and this must be suitably processed to protect individual privacy before the information can be shared further. The key difficulty is that there are major challenges in finding effective methods to deidentify free-text on a scale that does not damage data utility as a by-product.

Methods from natural language processing (NLP) have been developed to automatically scan clinical free-text data to identify potential identifiers, such as patients’ names, significant dates, and their family members. There are generally 2 approaches to automated deidentification. In a method referred to as *blacklisting*, deidentification algorithms remove identifying information so that these variables are masked in the data extract. Alternative methods focus only on isolating the relevant clinical information from personal identifiers via extraction of specified variables such as medication dosage instructions or diagnoses, which are *whitelisted* and preserved in text. Whitelisting can be thought of as the converse of blacklisting in that it extracts clinically informative data rather than excluding disallowed pieces of information [6-8].

**Figure 1:**
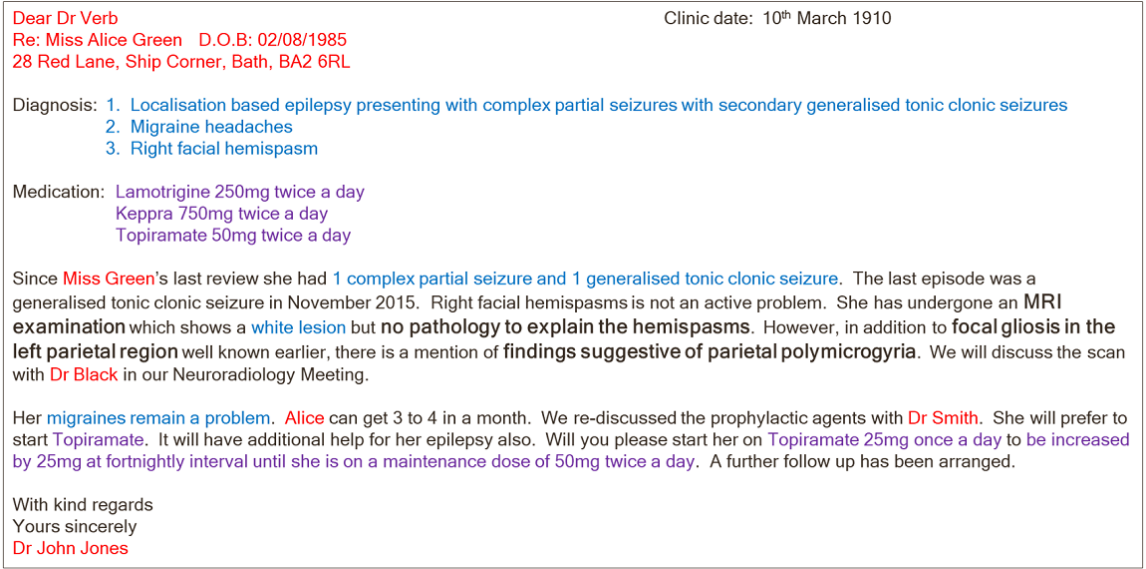
Synthetic clinic letter.

Each method has advantages and disadvantages and many permutations within them. For example, blacklisting can result in some personal identifiers remaining within deidentified text or important nonidentifiable data being removed from the text if the process is too stringent. Conversely, whitelisting can result in valuable data being left behind or false information being extracted inadvertently. Whitelisting also relies on a data extraction algorithm to choose the relevant clinical information, if no human is to see the identifiable data. A synthetic clinic letter is shown in Figure 1 to illustrate some of these challenges. As can be seen, a clinic letter can contain a variety of identifiable information about the patient and their care providers. Accuracy and the resulting privacy and utility in preparing free-text extracts for reuse depends on the sophistication of the methodologies in use. We provide some simple example problems for illustration purposes. Blacklisting could result in some personal identifiers remaining within purportedly deidentified text, for example, names that are also common words—Green and Verb. Conversely, whitelisting can leave valuable data behind, for example, *parietal polymicrogyria* without the word *suggestive* or *pathology to explain the hemispasms* without extracting the word *no*. There is much natural language processing and other methodological work underway to enable the creation of extracts that retain data utility and protect privacy. It is important to note that deidentification and extraction algorithms do not work *out of the box* but often have to be built and tested on specific data annotated by domain-specific experts to train and develop the algorithms. In general, algorithms can be trained to work to a standard comparable with that of a human annotator, but accuracy can decrease with increasing information complexity [6-8].

Being able to create free-text extracts that do not contain identifying information with a high degree of confidence is a fundamental issue but is not the only consideration for the governance of clinical free-text data. In addition, there are issues such as regulatory approvals including independent ethics committee review, working with or within the health service, accessing identifiable data for the deidentification and extraction process, engaging with clinicians to understand clinical concerns and point of care issues, patient involvement, questions around patient consent, retaining the ability for data linkage, and conditions and environments for data reuse.

### Main Aim

In recognition of these challenges, the main aim of this study was to develop recommendations for the creation of data governance standards to integrate with existing frameworks for personal data use, to enable free-text data to be used safely for research for patient and public benefit. As such, it will form a position paper and inform further work. In this context, we define *safely* as where the identity of the individual is highly unlikely to be discovered. We refer to this study as TexGov. It focuses on data governance and health services within the United Kingdom, but the main findings are applicable more widely.

## Methods

### Study Design

TexGov was designed to cover a variety of activities to develop data governance standards for using clinical free-text data in research. It included an outline of the UK data protection landscape for context, a rapid literature review on governance aspects in previous UK research studies using clinical free-text data, UK case studies with systems providing access to free-text data for more in-depth information, engagement with researchers to explore barriers and solutions in working with free-text data, and engagement with the public for views on socially acceptable approaches. These activities are described in the following sections.

### Data Protection Landscape

We reviewed the relevant legislation, regulations, and official guidance in place in the United Kingdom for context on governance aspects for the use of free-text data. This included the UK Data Protection Act (DPA) 2018 [9], the European Union General Data Protection Regulation (GDPR) 2016 [5], the Human Rights Act [10], the common law duty of confidentiality (CLDC [11]), the Caldicott Principles [11], and the Information Commissioner’s Office guidance on data sharing and anonymization [12,13].

### Free-Text Data Governance Practice

We gained information on data governance in working with clinical free-text data by carrying out a rapid literature review [14] and mini-case studies of systems making deidentified free-text data available for research in the United Kingdom. The methodology for the literature review is given below:

#### Search Strategy

Searches for studies indexed in PubMed and Web of Science (WoS) were conducted on January 15, 2019, using the following search terms: (1) “electronic health records” or “electronic medical records” or “electronic patient records” or “hospital records” or “personal health records” or “computerized patient records” or “computerized medical records” combined with (2) “text mining” or “natural language processing” or “free text” or “narrative.” Given our sole interest in ethical and governance procedures in the United Kingdom, the search was then restricted to papers using UK databases containing free-text data by using the following terms: “UK” or “United Kingdom” or “Britain” or “England” or “Wales” or “Ireland” or “Scotland.” The search was limited to human studies published in English. No restrictions were imposed on the year of publication.

#### Eligibility

To be eligible for this review, published research had to meet the following criteria: primary research using free-text records; UK-based health databases or data from UK hospitals; and information extracted from the text of (human, not veterinary) electronic medical records, medical letters or medical reports, and methods papers (eg, development or description of search and analytic tools), but only if the paper used patient data in the study.

#### Data Extraction

Characteristics of the included studies were extracted by one researcher and reviewed by another researcher and were as follows: (1) year published and lead author institution, (2) broad purpose of the study, (3) what and where data were accessed, (4) focus of free-text data, (5) whether the data were accessed in identifiable form, (6) whether the study used the health records in conjunction with other data, (7) main finding or conclusion, and (8) any ethics and governance detailed. We will focus on data governance aspects only, rather than the clinical value of using free-text data.

#### Case Studies

The case studies were conducted through face-to-face or telephone interviews in February 2019 and were structured to capture information about the main model for making data available, types of data included, data linkage capability, free-text deidentification or extraction method, and governance approvals. Notes were taken during the interviews and subsequently checked for accuracy with each interviewee.

### Engagement With Clinical Text-Mining Researchers

We held a 1-day workshop (January 16, 2019) for clinical free-text mining researchers to explore their perceptions of barriers and solutions in working with free-text data for sharing outside the clinical setting. Participants were recruited via advertisements on the Healtex website. The workshop was attended by 44 people and included outlines of the TexGov study aims, the findings of a previous citizens’ jury on using clinical free-text data for research [15], and data protection. These were followed by presentations from the NLP community on their approaches to working with clinical free-text data, during which the audience was asked to write down the topics to discuss in more detail. The identified topics were grouped into 4 themes and used in group discussions on challenges and solutions: (1) patient involvement at identifiable and deidentified data stages, (2) opt-in/opt-out consent models for the reuse of free-text data, (3) working with identifiable data for NLP algorithm development, and (4) deidentification methods and thresholds of reliability. Delegates were randomly allocated to one of the groups but were allowed to change to another if they felt strongly about a particular topic. Each of the discussions was facilitated and noted to capture views on the nature of the challenge, what can be done to address it, and how that can be achieved.

### Engagement With the Public

We discussed the TexGov study with 2 public groups. The first event was advertised to the general public and patient interest groups by the Alan Turing Institute and held at their premises on March 28, 2019, attended by approximately 50 people. It included a presentation on the TexGov study and a role-play exercise to illustrate how clinical free-text data are collected at a hypothetical general practitioner (GP) consultation and to engage the audience in discussion. It also included a panel discussion so the audience could ask questions to the representatives from the National Data Guardian’s office [16]; Understanding Patient Data, an independent patient data taskforce [17]; use MY Data, a movement of patients, carers, and relatives in support of using patient data [18]; and National Health Service (NHS) England [19]. Notes were taken during the panel discussion. This was followed by facilitated and noted group discussions on the topics of (1) Transparency and patient choice in the use of free-text data: what information do patients need and which are the best methods of dissemination? (2) Identifiability, deidentification, and anonymization: what is the best approach to making data available? and (3) Data access models and security in working with free-text data: how to balance restricting and facilitating access to data? These topics were prioritized from issues for discussion identified by registered delegates in advance of the workshop.

The second public engagement activity was conducted with the consumer panel based at Swansea University on May 1, 2019. This panel is made up of 18 members of the general public, and it advises on developments in the use of person-based data for research, such as via the Secure Anonymised Information Linkage (SAIL) Databank [20]. Following a description of the TexGov study and the preliminary findings, there was an open discussion so that members of the panel could provide their views on the work and the publicly acceptable way forward for the reuse of free-text data. Notes were taken on the views of the panel members.

## Results

### Data Protection Landscape

The Human Rights Act provides overarching legislation, with Article 8 setting out rights to enjoy a private life free of intrusion and interference, subject to restrictions in accordance with law in a democratic society [10]. Our review of UK statutory data protection law in the DPA [9] and the GDPR [5] showed that free-text data are not singled out by these instruments: the provisions apply to free-text data in the same way as any general person-identifiable data (PID) and special category health data. Similarly, free-text data given in confidence, such as in a physician-patient consultation, are subject to the same CLDC [11] principles and professional practice as for structured, coded data. The Caldicott Principles [11] set out fundamental good practices for the protection of information that could identify an individual but also highlight that data sharing can be as important as the duty to protect confidentiality. Within these principles and in the Information Commissioner’s Office guidance on data sharing and anonymization, there is nothing specifically stated about free-text data (Table 1). The requirements are for data use to be subject to an appropriate lawful basis and for data to be processed to protect privacy before secondary uses, unless another justification (such as participant consent for research) is in place. This is a challenge for the reuse of clinical free-text data: the difficulty in being confident (and providing evidence) that deidentification is adequate when making claims of anonymity or minimizing the use of personal data, so that information derived from the unstructured format of free-text data can be safely taken forward for reuse.

**Table 1:** Summary of relevant data protection landscape.

Item	Key points
UK Human Rights Act	Sets out an individual’s rights to enjoy a private life free of intrusion and interference
European Union General Data Protection Regulation and the UK Data Protection Act	Provisions for processing general person-identifiable data and special category health data
Common Law Duty of Confidence	Governs the use of data given in confidence, such as in a physician-patient consultation
Caldicott Principles	Set out good practice for the protection of information that could identify an individual and the importance of data sharing
Information Commissioner’s Office	Provides guidance on data sharing and anonymization

### Data Governance

The search identified 45 papers from PubMed and 340 papers from WoS, with 354 papers remaining after the removal of duplicates. After screening abstracts and full texts, 51 studies were defined as eligible for inclusion in the rapid review. Medical records were accessed via 3 main databases: (1) the Health Improvement Network (THIN) primary care database [21,22] (N=7) [23-29], (2) South London and Maudsley NHS Foundation Trust (SLaM) with clinical records accessed via the Clinical Records Interactive Search (CRIS) system [30-32] (N=16) [33-49], and (3) the Clinical Practice Research Datalink (CPRD) [50] (N=13) [51-62]. One study used the SAIL Databank [63]. There were 13 independent studies, which were not associated with a data management infrastructure [64-76].

The THIN and CPRD databases contain NHS patient primary care records, whereas the CRIS system includes clinical records for people who have used a range of NHS mental health services and substance misuse services in the United Kingdom. The study that used the SAIL Databank accessed the primary care dataset. The independent studies accessed a range of free-text records, including from accident and emergency department, outpatient department, intensive care, primary care, and prescribing datasets. All records in the studies that used THIN, CRIS, CPRD, and SAIL Databank were anonymized, and no personal information was, therefore, available to researchers. The independent studies were more variable depending on specific approvals and permissions, for example, identifiable data accessed by authorized hospital staff within an NHS organization [64,71] or data extracted following the ethical approval of the study [76].

All studies reporting the use of THIN noted ethical approvals, and 3 studies also cited the overarching THIN data resource approvals by the NHS South East Multicenter Research Ethics Committee. All but one [48] of the studies that used the CRIS data resource noted approval by the Oxfordshire research ethics committee and the service user-led oversight committee study approval, which must be granted before access to the anonymized data is permitted. Of the 13 studies using CPRD free-text data, 4 provided no ethical approval details [51-53,61]. All the remaining studies in this group detail some level of ethical approval, either study approval or the resource multicenter research ethics committee approval for all observational research using CPRD data. The study accessing free-text data in SAIL Databank does not cite the information governance review panel (IGRP) approval that must have been sought before access to SAIL data was granted [63]. Of the 13 independent studies, 7 detailed the local ethical approval process [67-69,72,73,75,76]. One stated that ethical approval was not necessary, as the purpose of the study was to measure service provision retrospectively without using identifiable patient information [74], and the remaining 7 studies did not mention ethical approvals [64-66,70,71].

None of the 7 studies that used THIN detailed the data access model, which could have been via a sublicense agreement enabling access for a defined period to conduct unlimited studies, subject to protocol approval, or release of data extracts consisting of subsets of raw data in accordance with researchers’ study protocols and specifications. CRIS is accessed within the NHS system by researchers at the Maudsley Biomedical Research Centre; as such, most lead authors (or at least one coauthor) of the 16 studies using SLaM clinical records were based at King’s College Hospital. Some, but not all, studies specify this access route. CPRD (formally known and referenced in studies as the General Practice Research Database) operated on a data release model under the strict CPRD and data control terms. Few of the retrieved studies specified this model. The study using free-text records in the SAIL Databank did not detail the access route, that is, following necessary governance approvals and safe researcher training, data can be accessed remotely within the safe haven with no external data release [77]. The 13 independent studies provided very little information on how data were accessed. A summary of key information from the rapid review is given in Table 2.

**Table 2:** Key information from the literature review.

Institute	Study	Free-text search	Linkage to additional datasets	Use of identifiable data	Ethical approval
THIN^a^ (n=7)	[23-29]	[23] cause of death; [24] ischemic cerebrovascular events; [25] diabetic retinopathy and diabetic maculopathy diagnoses; [26] child maltreatment; [27] colorectal cancer recording; [28] endometriosis diagnosis; and [29] major malformations and comments assigned to referrals to specialists	Yes: [23,25,27,28]; no: [24,26,29]	Yes: [26]; all others: no	THIN data resource approved by NHS South East Multicenter Research Ethics Committee
CRIS^b^ (n=17)	[33-49]	[33] smoking status; [34] extrapyramidal side effects and adverse drug events; [35] antipsychotic polypharmacy; [36] negative symptoms of schizophrenia; [37] mood instability; [38] cannabis use; [39] medication descriptions; [40] characteristics of people with Alzheimer disease; [41] delivery of cognitive behavioral therapy for psychosis; [42] notes and correspondence on diagnoses of mental disorders; [43] adverse drug events; [44] symptoms of severe mental illness; [45] risk factors for depression; [46] presence of negative symptoms and antipsychotic use; [47] suicide ideation and attempts; [48] information on hepatitis C and HIV; and [49] registered company addresses	Yes: [39,42,45]	No	CRIS approved for secondary analysis by the Oxfordshire Research Ethics Committee. A service user-led oversight committee considers all proposals before access to the anonymized data is permitted
CPRD^c^ (n=13)	[51-62]	[51] heart defects; [52] congenital malformations; [53] pregnancy outcomes; [54] ovarian cancer diagnoses; [55] cause of death; [56] coronary angiogram results; [57] keywords for rheumatoid arthritis; [58] markers for rheumatoid arthritis; [59] disease-modifying antirheumatic drugs; [60] records of visible hematuria, jaundice, or abdominal pain; [61] drug usage values and administrations; and [62] terms indicating allergic bronchopulmonary aspergillosis cases	Yes: [51-53]; no: [54-62]	No	Multicenter research ethics committee approval was in place for all observational research using CPRD data
SAIL^d^ (n=1)	[63]	[63] symptoms of ankylosing spondylitis	No	No	Ethical approval is not required for the use of anonymized data within SAIL. An independent information governance review panel assesses all proposed uses of SAIL data.
Independent studies (n=13)	[64-76]	[64] string “asth” for asthma; [65] range of clinical terms; [66] reason for admission; [67] test results for stages 3-5 chronic kidney disease; [68] search in reports of CT^e^ scan of brain for stroke; [69] search in reports of CT scan of brain for stroke, subarachnoid hemorrhage, or ischemic stroke; [70] keywords on hearing aid decisions; [71] reasons for dose omissions; [72] reasons for deaths due to unsafe care; [73] breathlessness and wheeze symptoms; [74] focal liver lesions; [75] cardiology information; and [76] clinician discourses compared with patient narratives	Yes: [68,69,76]; no: [64-67], [70-75]	Yes: [64,71,73,76]; no: [67-69,72,74,75]; and not clearly stated: [65,66,70]	Variable depending on particular study

^a^THIN: The Health Improvement Network.

^b^CRIS: Clinical Records Interactive Search.

^c^CPRD: Clinical Practice Research Datalink.

^d^SAIL: Secure Anonymised Information Linkage.

^e^CT: computed tomography.

Table 2 summarizes the key information reported in the studies included in the rapid literature review. The first column shows the number of studies relating to each institute. The study numbers in subsequent columns are as per the reference list (and are not counts of studies). The extent to which individual studies reported approvals was variable, although (at least for those attached to THIN, CRIS, CPRD, or SAIL) they would have had to abide by the mechanisms in place before data access was granted because access is under institutional control. Rather than attempting to include all details by study, we showed the standard approvals in the final column with fuller information in the text.

The interviews provided further information on systems providing access to data derived from clinical free-text sources. The purpose was to learn about their methods and from their experiences. We do not describe the models in full detail, as that would be beyond the scope of this paper. The THIN database was created in 2003 by In Practice Systems (Vision). THIN is a primary care data repository with the capability to extract free-text as well as structured data from GP practices, but currently, only structured data are released to external researchers. THIN has collaborated with University College London (UCL) to promote academic use of the data. As part of this, a copy of the THIN database will sit in a UCL data safe haven, which can be accessed by UCL researchers. THIN is seeking ethical approval to enable the use of the free-text data for academic research within UK academic or NHS safe havens. Free-text data will be subjected to an automated deidentification process using a blacklisting method. The full data governance model at UCL is in the final stages of development at the time of writing. THIN is overseen by an advisory committee involving patient, clinician, and researcher representatives, and all studies are reviewed by a scientiﬁc review committee before data access can be granted [21,22].

CRIS is a medical record inquiry program that has been implemented in a number of mental health trusts. Our example relates to SLaM, and so, we refer to *CRIS at SLaM* because other NHS Trusts might not operate CRIS under exactly the same principles. Data are held in a repository inside the Trust firewall (ie, in the same domain as the original health record), which comprises a database of structured and free-text mental health records that are deidentified, but linkable at the individual level, before entering the repository. Unlike some blacklisting algorithms that rely on a lexicon to ascertain and remove names, the CRIS deidentification algorithm has access to individual health records and makes reference to this to blank out the name of the patient. CRIS at SLaM has ethical and s251 approval: the latter for occasions when PID need to be exported outside the firewall to enable linkage. It also has approval from the Trust Caldicott Guardian and the executive board. The person accessing the data in the safe haven must have a SLaM contract, a SLaM honorary contract, or a research passport (an accreditation issued by the Health Research Authority [78]). All projects must include at least one member of the study team who has an honorary or substantive SLaM contract and can act as a guarantor [30-32].

SAIL is a repository that holds multiple health and wider administrative datasets in a deidentified form about the population of Wales. SAIL Databank does not process PID but uses a trusted third party to receive the PID and carry out a matching and deidentification process, with the creation of a consistent identifier unique for each person represented to enable individual-level linkage across datasets. Further controls are enacted to provide access to data for research in anonymized form within a safe haven. The SAIL data comprise extracts of structured, coded data, with minimal free-text data at present. However, work is underway to incorporate free-text data so that they can be included in studies when needed. Unlike approaches that deidentify free-text data (blacklist) and incorporate the remainder, the algorithm used by SAIL extracts medical terms and descriptors (whitelist) at the NHS source for incorporation so that only nonidentifiable structured data leave the organization. SAIL Databank took this approach because of the risks of introducing insufficiently deidentified information into the databank. Working with free-text data at source is by means of an NHS honorary contract [8], and all proposals to use SAIL data must have received approval from an independent IGRP before access can be granted via the data safe haven [77].

CPRD operates on a data release model and used to collect and release deidentified (blacklisted) free-text data under strictly controlled protocols. However, this was discontinued in 2013 on advice from the Information Commissioner’s Office, and the catalog of free-text data was destroyed. At present, CPRD has no plans to develop NLP or an automated text anonymization service. Instead, they liaise directly with GPs on behalf of researchers to validate codes to supplement coded data with additional information.

### Engagement With Clinical Text-Mining Researchers

A summary of the information gained during the discussions on each theme is shown below in relation to the nature of the challenge, what could be done to address it, and how this could be achieved.

#### Patient Involvement at Identifiable and Deidentified Data Stage

This theme relates to the principles of engagement with the public for good research practice in general.

##### Nature of the Challenge

Delegates felt that there was a challenge in balancing what could be perceived as a lack of individual choice in nonconsented data cohorts with the risk of selection bias in consented cohorts, particularly if there is doubt about the level of knowledge among individuals, including conceptual differences between coded and free-text data.

##### What Can Be Done

Additional public engagement in free-text research was seen as needed. This should be linked with wider information about patient data uses to help people better understand the issues and gain their input. This could contribute to a checklist of recommendations for researchers using free-text data, so the public views on the use of free-text data are taken into account.

##### How It Can Be Achieved

Public panels and existing groups can help clarify reasonable expectations regarding the use of free-text data. It would be useful to have a repository of findings from research using free-text data showing its clinical benefits. This could also include information in lay format on advances in methodological research to improve processes of handling free-text data.

#### Opt-In/Opt-Out Consent Models for Reuse of Free-Text Data

This theme relates to managing individual choices in the use of free-text data for research.

##### Nature of the Challenge

Discussions focused on the need to convey a reasonable understanding of the motivations, risks, and benefits of researchers accessing free-text data and how data reuse is governed. In particular, the challenge of providing appropriate consent options was highlighted.

##### What Can Be Done

As consent might not be required, or even be possible, for processing large-scale free-text data across populations, delegates stressed the need for guidance and good practice on free-text data reuse in line with governance requirements and expectations.

##### How It Can Be Achieved

A wider examination of existing practice used by government departments along with clinical and regulatory codes of practice would provide further guidance on how to manage consent options. Possible solutions could be piloted and evaluated for practicality.

#### Working With Identifiable Data for Natural Language Processing Algorithm Development

This theme relates to the practicalities in accessing free-text data in identifiable form to prepare extracts for research.

##### Nature of the Challenge

Free-text data are needed in their original (raw) form for effective NLP algorithm development and maintenance, yet the data are identifiable, and some contain sensitive information. In addition, the free-text datasets accessed need to be of sufficient scale to avoid introducing bias when developing NLP methods.

##### What Can Be Done

There is a need to clarify whether enabling data access via a safe haven can provide a suitable solution, if patient data can be used without consent for the purpose of training algorithms, and the usefulness of publicly available datasets.

##### How It Can Be Achieved

Delegates suggested possible solutions by creating a *Text Bank* to support NLP development via data donation, the use of synthetic datasets, and sharing annotated free-text data with other developers.

#### Deidentification Methods and Reliability Thresholds

This theme relates to residual reidentification risk and data utility in the datasets used for research.

##### Nature of the Challenge

Delegates focused on the challenges in quantifying the residual risk of reidentification in free-text data extracts, particularly in the face of the high degree of data variability and the absence of a gold standard threshold for free-text deidentification.

##### What Can Be Done

Discussions highlighted the need for robust security with only trusted access and an audit trail, and that free-text data extracts should be treated as potentially identifiable. More research on the relationship between the accuracy of the deidentification algorithm, risk of reidentification, and data utility was identified as a need.

##### How It Can Be Achieved

Accessing free-text data extracts only within safe havens by small teams embedded within organizations, and with clear lines of accountability, was seen as a positive step. A range of risk models should be employed to avoid overscrubbing the data with consequent loss of data utility and to determine acceptable levels of deidentification.

### Engagement With the Public: Panel Discussions at the Turing Institute Event

The panel discussion at the Turing Institute event with representatives from the National Data Guardian’s office, Understanding Patient Data, use MY Data, and NHS England raised some important points. The National Data Guardian representative highlighted that data protection legislation is not a barrier to data sharing, but there is a need for proper transparency and trustworthiness to build public confidence. In addition, there is a need for a joined-up approach in developing consistent standards, language, and message. The representative from understanding patient data stressed the huge potential in being able to use clinical free-text data beyond direct care but also highlighted the challenges in being able to enact this in an acceptable way, respecting privacy and ensuring accuracy in data extracted. They also spoke of the need for evidence of the potential benefits to patients of the use of their clinical free-text data. The representative from use MY Data emphasized the enormous potential of, as yet, largely underutilized free-text data for research purposes, seeing this as an *atrocious waste*. This was accompanied by an expression of the need for ongoing patient engagement and that use MY Data is an instruction, not a request, as the group wants greater use of patient data and is working hard to convey this message to decision makers. The NHS England representative spoke about the CLDC and the need for free-text data use not to impact the confidentiality of medical consultations. All the panelists emphasized the need to demonstrate to the public the benefits of using clinical free-text data and the importance of working in accordance with existing research and information governance frameworks, but that adaptations to these frameworks may be required for emerging data types and formats.

### Engagement With the Public: Group Discussions at the Turing Institute Event

A summary of the points emerging from the group discussions with delegates at the Turing Institute event is provided in relation to knowledge gaps, involvement and engagement, and suggested solutions.

#### Transparency and Patient Choice in the Use of Clinical Free-Text Data: What Information Do Patients Need and Which Are the Best Methods of Dissemination?

##### Knowledge Gaps

The delegates acknowledged the efforts that go into public involvement and engagement but highlighted the seldom heard voices, such as hard to reach groups, marginalized groups, and young people.

##### Involvement and Engagement

They felt that because of risks of misunderstanding, information for the public should be layered, that is, it should be contextualized by founding it on health data and research before adding the particular features, risks, and benefits of free-text data.

##### Suggested Solutions

Information should be provided in plain language and in accessible formats, taking into account differing needs and abilities. Delegates recommended tapping into patient networks as authentic, credible ambassadors for discussions with the wider public and the value in working with communication experts. It was seen as important to explain that although there are challenges in effectively deidentifying free-text data, it is a format of data and not fundamentally different from other health data.

#### Identifiability, Deidentification, and Anonymization: What Is the Best Approach to Making Data Available?

##### Knowledge Gaps

The delegates felt unclear about how the GDPR (introduced in 2018) would apply within the context of the existing UK regulations and uncertainties around how best to make data available and minimize risks. There were uncertainties around how one might seek informed consent to participate in research in a way that participants would understand but also how wider public would be able to grasp concepts in the abstract.

##### Involvement and Engagement

The delegates expressed the need for clearer information on the relationship between having a lawful basis for data processing under the GDPR and the fact that this does not negate the need for consent to participate in research. With regard to identifiability, delegates felt that the research community should make a strong case for the use of free-text data in research, including that it was important not to lose that richness.

##### Suggested Solutions

Delegates felt that engagement and educational events should be carefully planned in terms of how they could empower people to make informed decisions when it came to reusing of free-text data. This would need to provide a compelling reason for research to look at minimally deidentified free-text data to preserve the richness, balanced against how the risks to participants could be managed.

#### Data Access Models and Security in Working With Free-Text Data: How to Balance Restricting and Facilitating Access to Data?

##### Knowledge Gaps

Delegates acknowledged a general lack of awareness of different data access models and the degrees to which data can be controlled, shared, or made openly accessible. They stressed the risks of not using the free-text data and the benefits that could be added to research.

##### Involvement and Engagement

Delegates felt that there should be more involvement and engagement with the public to explain due diligence processes and safeguards in managing free-text data. This should include interactive methods and demonstrations, not limited to passive information transfer.

##### Suggested Solutions

Delegates stressed that free-text data need to be used so that the United Kingdom does not lag behind other countries. The research community should be open to the public and explain that deidentification of free-text data may be imperfect. Patient groups should follow the example of use MY Data and lobbying data controllers for free-text data to be made more accessible to researchers.

### Engagement With the Public: Consumer Panel

Having been presented with the findings of the previous engagement activities (described here), the consumer panel agreed and furthermore suggested that patient involvement should be included at all stages practical in the development of algorithms to avoid bias in relation to diversity; that involvement should be ongoing because of societal attitudinal changes over time and as new knowledge comes to light; and that a databank of clinical free-text data for algorithm development would be a good thing, provided that it is done transparently and properly managed.

## Discussion

### Principal Findings

To our knowledge, this is the first study to explore the data governance aspects for using clinical free-text data and to do this through a combination of outlining the legislative and regulatory backdrop, reviewing literature and systems using free-text data extracts in health research, and gaining the perspectives of free-text data researchers and members of the public. In terms of UK data protection legislation and guidance, there is nothing specific in relation to free-text data. In many ways, this was to be expected, as legislation tends to be high level needing interpretation and justification of an appropriate lawful basis on how data are to be used. In addition, free-text is a data format and not a different type or category (eg, health, political views, and ethnicity) of data, and so, it is covered by the data protection provisions for health data. In terms of official guidance, such as from the Information Commissioner’s Office, the need to properly manage and process all personal data for reuse is addressed in codes of practice [12,13]. However, until recently, the vast majority of routine health data made available for secondary use was from coded records. As such, clinical free-text data are among the emerging research datasets, such as genomic and imaging data, and warrant further attention to develop official guidance to simultaneously safeguard individual privacy and maximize data utility for research in the public interest.

Our literature review revealed a variety of published studies that used data derived from free-text sources (eg, clinical notes and referral letters), sometimes in conjunction with coded health records and other datasets. The majority of these were conducted through established systems (eg, THIN and CRIS) specializing in making clinical free-text data available for approved research, in accordance with their operating and governance models. There were also some studies not associated with one of these systems. In many cases, the authors properly reported on how data were accessed and provided information on regulatory and governance approvals in place. However, there was also inconsistency and a lack of information in some studies, although this might reflect reporting rather than practice [79]. It is not always easy to ensure that authors provide governance details, but at least in studies from the established systems, this is a matter for publication policies that are part of their good governance practice. However, it should also be addressed by journal editors to ensure that there is a section providing transparency on methodology and data governance where free-text data (or person-based data) have been used.

There are a variety of automated methods to deidentify or extract data from free-text clinical documents for reuse in research or other purposes beyond direct clinical care. Our review showed that blacklisting methods are used by THIN and CRIS (and previously used by CPRD) and whitelisting methods are used by SAIL. THIN and SAIL operate in the higher education sector, whereas CRIS operates behind an NHS Trust firewall. All 3 institutes operate a data safe haven model, rather than external data release, and all have data governance models in place. From engagement with clinical text-mining researchers, it is clear that although the state-of-the-art systems achieve performance comparable with manual deidentification, there are still uncertainties about the efficacy of blacklisting methods and acceptable thresholds of reliability. Further work is needed to address this issue. However, whitelisting is not a panacea, as it also relies on algorithms that must be tested on identifiable data and can risk over- or underextracting data.

We have noted that using only small samples of identifiable free-text data sources can lead to bias. The creation of a free-text databank donated on a voluntary basis for developmental work would help to advance this field of work. At the same time, there is a need to minimize the need for human access to clinical free-text data through continual improvement in technological NLP methods for deidentification of clinical narratives. Further developments are also needed in other areas of text processing so that free-text data can be more efficiently converted to a structured format before reuse. Although this is advantageous in terms of risk minimization, it risks the loss of information richness that could be needed for qualitative research, exploring areas such as language, culture, and patient stories in the full free-text data, and reduces the future possible uses of the text data, with new extractions needed for subsequent research studies.

The clear need for patient involvement and engagement in the development of reusing free-text data was highlighted in the events with researchers and the public. This included a call for public-facing activities to be ongoing, inclusive, and transparent. Public delegates at the Turing Institute event and the consumer panel were generally positive about using free-text data for research, and this is in line with previous work with the Brighton Citizen’s Jury (in 2018). This jury suggested that people’s concerns could be mitigated with comprehensive patient-facing information about how, when, and under what conditions patients’ free-text data might be used for research. Furthermore, patients should be involved as key stakeholders throughout all stages of the research process. The public also expects that researchers are committed to a culture of continuous improvement of methods for coding, anonymizing, processing, and safeguarding clinical free-text data [15].

Participant consent was a theme discussed at the clinical text-mining researcher workshop and again at the Turing Institute event, as it was at the Brighton Citizens’ Jury. There was a recognition that it would not generally be practicable to seek opt-in consent to incorporate deidentified free-text extracts into data safe havens for reuse in research. The possibilities of opt-out consent were also explored and were considered favorable if they could be enacted in accordance with existing patient data guidelines. In England, there is a national opt-out where patients can choose not to have their data used for purposes beyond their direct care. However, in practice, this only applies to situations where the reuse of the data relies on s251 approval to set aside the CLDC [80]. As such, it is not a comprehensive opt-out mechanism and is not equipped to allow someone to specify a particular data format. Working in Wales, SAIL Databank operates its own opt-out mechanism by allowing individuals to inform their GP that they do not wish their data to be provided to the databank; again, this is an all-or-nothing option. More granular optouts would present difficult challenges in defining data content for inclusion and exclusion. Nevertheless, the mechanisms in place do provide individuals with choices they can exercise in relation to their patient records.

The panel discussion at the Turing Institute event stressed the importance of operating within existing frameworks for all data, not singling out clinical free-text data as fundamentally different. Although challenges remain for meaningful opt-out consent, mitigation can be provided via clear privacy notices, lawful bases for data processing, and transparent information on safeguards and their limitations. However, although the group discussions highlighted the great importance of being able to make good use of clinical free-text data, we are still left with the situation that the deidentification (blacklisting) of free-text data is imperfect, and extracting only certain elements (whitelisting) produces only a study-specific data extract. Although established data systems making free-text extracts available in one form or another have data governance controls in place, there are cases where further measures are needed at the project level. Two examples are studies that are not associated with established systems such that the data might be subject to external release (ie, to be held outside a data management infrastructure) and studies that need to use free-text data for qualitative analysis, where having the data translated into coded form would not be adequate.

### Recommendations

In addition to good practice in handling person-based data in general, we propose the following set of recommendations and suggestions for further work to operate in accordance with, and augment, existing research and information governance frameworks:

- There is a need for clear regulatory guidance on data governance for the reuse of clinical free-text data, taking into account factors including whether the data are deidentified through blacklisting or extracted via whitelisting; the native data are housed at their original source or safe havens or exported to external users, and the extracts are in a coded or raw free-text form.
- Patients and the public should be better informed about free-text data flows and uses, including the availability of opt-out arrangements for the reuse of patient data for research in accordance with jurisdictional policies. This requires commitment from researchers and the public.
- The use of free-text data and results of studies should be publicized to all stakeholders, but particularly patients, so that the public can see that free-text data can bring additional benefits. This could take the form of regularly updated case studies in a central location, including information about the data used and the findings of the study.
- Further improvements are needed in deidentification and extraction algorithms with a better understanding of the relationships between the accuracy of deidentification and thresholds of reidentification risk. This could be facilitated by a national challenge with public demonstrations on how free-text data are deidentified.
- Owing to the current uncertainties, blacklisted clinical free-text data should be treated as potentially identifiable and access for research restricted to accredited data safe havens, unless a thorough review is conducted before release.
- Organizations providing access to clinical free-text extracts should stipulate in their publication policies a data governance statement to be placed in publications, and journals should require a suitable statement for all studies using data derived from free text.
- The specifics around the creation of a databank of donated clinical free-text data to support the construction of deidentification and extraction of algorithms should be explored; this would necessarily include further public engagement and government and NHS commitment.

### Limitations

We acknowledge that the TexGov study was subject to limitations. It was a small study of a few months in duration and, as such, could not be comprehensive in scope. It was limited to the United Kingdom, and the governance of data use may differ in other jurisdictions. However, we believe that the principles will be largely applicable beyond the United Kingdom. We were unable to engage specifically with data providers or clinicians who created the data to gauge their viewpoints. This could be a focus of future work.

### Conclusions

It is well known that clinical free-text data represent a rich resource for reuse in research, but that there are particular challenges in working with unstructured data to safeguard privacy and maximize data utility. We have shown that lessons can be learned from established systems providing access to data derived from clinical free text and that the views of text-mining researchers and members of the public provide valuable insights. We present the new knowledge gained in this unique study in the form of a position paper to work toward the development of data governance standards for the reuse of free-text data. While recognizing that free-text data are not fundamentally different from other patient data and the need to work within existing data governance frameworks, we propose that there is a need to develop the TexGov recommendations, with commitment and investment, to expand and assure the safe use of free-text data for public benefit.

## Abbreviations

CLDC: common law duty of confidentiality
CPRD: Clinical Practice Research Datalink
CRIS: Clinical Records Interactive Search
DPA: Data Protection Act
EHR: electronic health record
GDPR: General Data Protection Regulation
GP: general practitioner
IGRP: Information Governance Review Panel
NHS: National Health Service
NLP: natural language processing
PID: person-identifiable data
SAIL: Secure Anonymised Information Linkage
SLaM: South London and Maudsley NHS Foundation Trust
THIN: the Health Improvement Network
UCL: University College London
WoS: Web of Science
